# Role of microRNA-381 in bladder cancer growth and metastasis with the involvement of BMI1 and the Rho/ROCK axis

**DOI:** 10.1186/s12894-020-00775-3

**Published:** 2021-01-06

**Authors:** Dayin Chen, Liang Cheng, Huifeng Cao, Wensi Liu

**Affiliations:** Department of Urology, The First Affiliated Hospital of Jiamusi University, No. 348, Dexiang Street, Jiamusi, 154002 Heilongjiang People’s Republic of China

**Keywords:** miR-381, BMI1, Rho/rock signaling pathway, Bladder cancer, Microarray analysis

## Abstract

**Background:**

Emerging evidence has noted the important participation of microRNAs (miRNAs) in several human diseases including cancer. This research was launched to probe the function of miR-381 in bladder cancer (BCa) progression.

**Methods:**

Twenty-eight patients with primary BCa were included in this study. Cancer tissues and the adjacent normal tissues were obtained. Aberrantly expressed miRNAs in BCa tissues were analyzed using miRNA microarrays. miR-381 expression in the bladder and paired tumor tissues, and in BCa and normal cell lines was determined. The target relationship between miR-381 and BMI1 was predicted online and validated through a luciferase assay. Gain-of-functions of miR-381 and BMI1 were performed to identify their functions on BCa cell behaviors as well as tumor growth in vivo. The involvement of the Rho/ROCK signaling was identified.

**Results:**

miR-381 was poor regulated in BCa tissues and cells (all *p* < 0.05). A higher miR-381 level indicated a better prognosis of patients with BCa. Artificial up-regulation of miR-381 inhibited proliferation, invasion, migration, resistance to apoptosis, and tumor formation ability of BCa T24 and RT4 cells (all *p* < 0.05). miR-381 was found to directly bind to BMI1 and was negatively correlated with BMI1 expression. Overexpression of BMI1 partially blocked the tumor suppressing roles of miR-381 in cell malignancy and tumor growth (all *p* < 0.05). In addition, miR-381 led to decreased RhoA phosphorylation and ROCK2 activation, which were also reversed by BMI1 (all *p* < 0.05). Artificial inhibition of the Rho/ROCK signaling blocked the functions of BMI1 in cell growth and metastasis (all *p* < 0.05).

**Conclusion:**

The study evidenced that miR-381 may act as a beneficiary biomarker in BCa patients. Up-regulation of miR-381 suppresses BCa development both in vivo and in vitro through BMI1 down-regulation and the Rho/ROCK inactivation.

## Background

Cancer is a major public health concern across the globe, and the urinary bladder cancer (BCa) is the most common neoplastic diseases in the urinary system with 81,400 estimated cases in 2020 in the United States alone according to the up-to-date Cancer Statistics [1]. Nearly 165,000 cases die from this malignancy around the world every year, and males have a threefold predominance in morbidity against females, possibly because of the long-term smoking prevalence and exposure to occupational carcinogens [2]. BCa is allocated to two important branches according to the disease states, which are non-muscle invasive BCa (NMIBC, < T2 stage) and the more risky and metastatic type, muscle invasive BCa (MIBC, T2-T4 stage) with the current or future distant invasion potential [3]. However, tumor recurrence is a major challenge in BCa treatment with a rate of 61% in the first year, and approximately 3–15% recurrent NMIBC may progress to the invasive type MIBC when obtaining additional genetic mutations [4]. The current treatment for BCa is far below expectations, with the survival rate of patients seeing little improvement during the last decades [2]. Identifying more molecular mechanisms may help develop novel effective treatments for this disease.

MicroRNAs (miRNAs), a large family of non-coding RNAs with 22 nucleotides in length, regulate multiple biological processes and are aberrantly expressed in human diseases including cancers [5]. miRNAs are well-known to exert key functions by inducing complementary target mRNA repression post-transcriptionally through the 3′ untranslated region (3′ UTR) [6]. Here in the paper, the miRNA microarray analysis was performed with miR-381 found as a major down-regulated miRNA in the BCa tissues. miR-381 has been noted as a tumor inhibitor in many human cancers [7, 8]. In addition, it is noteworthy that miR-381-3p may inhibit CDK6 and MET-mediated epithelial-mesenchymal transition (EMT) and cell cycle progression of BCa cells [9]. But the roles of miR-381 in BCa progression are far away from being fully researched. Here, our study identified BMI1 as a putative mRNA target of miR-381. BMI1 is a key protein of polycomb repressive complex 1 that functions in integrity maintenance, and it has been increasingly known as a factor in the development of multiple human cancers [10]. Considering the potential downregulation of miR-381 in BCa and its anti-tumor effects reported in other malignancies, we hypothesized that miR-381 inhibits malignant behaviors of BCa through the down-regulation of BMI1. To identify this hypothesis, the expression profiles of miR-381 and BMI1 in BCa tissues and cells was determined. The binding relationship between miR-381 and BMI1 was validated through a luciferase assay. Altered expression of miR-381 and BMI1 was induced to explore their roles in BCa development in both cell and animal experiments, and the potential downstream signaling pathway was identified.

## Methods

### Clinical sample collection

BCa tissues and the paired adjacent tissues were obtained by tumor resection surgery from 28 BCa patients (22 males and 6 females) who were admitted into the First Affiliated Hospital of Jiamusi University from January 2013 to January 2015 with a median age of 63 years. A 4-year follow-up study was performed to record the prognosis of patients at a 3-month interval. The patients were divided into T1 to T4 stages according to the Tumor-Node-Metastasis (TNM) staging, with 7 in T1, 13 in T2, 5 in T3, and 3 in T4. All included patients had neither pre-surgery treatment history nor chemoradiotherapy history, and they were pathologically diagnosed as primary BCa free of other diseases. The tumor tissues and the adjacent tissues (at least 3 cm away from the tumor tissues) were resected during surgery and preserved in liquid nitrogen. The demographic characteristics of the respondents are exhibited in Table 1.

**Table 1:** Clinical feature of included patients

Item	Group	n
Gender	Male	22
	Female	6
Age	≤ 60	5
	> 60	23
TNM stage	T1	7
	T2	13
	T3	5
	T4	3
Tumor number	Multiple	10
	Single	18
Tumor size (cm)	≤ 3	18
	> 3	10
Lymph nodes metastasis	yes	8
	no	20

*TNM* Tumor Node Metastasis

### miRNA microarray analysis

Four patients in T1, T2, T3 or T4 stage (one in each stage) were enrolled for miRNA microarray analysis. The tumor and the paired adjacent tissues from patients were collected for total RNA extraction using TRIzol Reagent (Invitrogen, Carlsbad, CA, USA). Total RNA was concentrated in isopropanol, and then the RNA purity was detected using NanoDrop 2000C (Thermo Fisher Scientific Inc., Waltham, MA, USA) and the RNA quality was determined by formaldehyde denatured agarose gel electrophoresis. Then, 50 μg total RNA was purified using a Taqman miRNA ABC purification kit (Thermo Fisher) and hybridized with the beads. The beads were then washed with magnet, and the miRNAs were eluted 3 times to collect the miRNAs. Thereafter, the collected miRNAs were labeled using a miRNA Complete Labeling and Hyb Kit (Agilent, USA). In brief, 100 μg total RNA was incubated in 10 μg Labeling Spike-In at 37 °C for 30 min, treated with DMSO at 16 °C for 2 h, dried in a vacuum concentrator at 45 °C for 3 h, and then treated with Hybridization Mixture and Hyb labeling at 55 °C for 20 h of hybridization. Then, the miRNAs were further hybridized with Human miRNA Microarray Release 14.0,8 (Agilent, USA) and scanned using a SureScan Dx Microarray Scanner (Agilent, USA). The obtained data were subjected to Quality Center analysis and normalization to produce the heatmap for differentially expressed miRNAs.

### Reverse transcription quantitative polymerase chain reaction (RT-qPCR)

Total RNA from cells or tissues was extracted using the TRIzol Reagent and reversely transcribed into cDNA using a PromeScript^TM^RT Master Mix Kit (Takara Bio, Jan). Relative gene expression was quantitated using a SYBR®Premix Ex Taq™II Kit (Takara Bio, Jan). The qPCR was performed on a Mx3005P System (Stratagene, USA). Quantification of each sample was repeated by three times. Relative expression was evaluated using the 2^−ΔΔCt^ method with U6 and GAPDH as internal references. Table 2 exhibits the primer sequences.

**Table 2:** Primer sequences for RT-qPCR

Gene	Primer sequence (5′–3′)
miR-381	F: TATACAAGGGCAAGCTCUCTGT
	R: TGCGGGTGCTCGCTTCGGCAGC3
BMI1	F: TGGATCGGAAAGTAAACAAAGAC
	R: TGCATCACAGTCATTGCTGCT
GAPDH	F: GGGAGCCAAAAGGGTCATCA
	R: TGATGGCATGGACTGTGGTC
U6	F: CTCGCTTCGGCAGCACA
	R: AACGCTTCACGAATTTGCGT

*RT-qPCR* reverse transcription quantitative polymerase chain reaction, *miR-381* microRNA-381, *GAPDH* glyceraldehyde-3-phosphate dehydrogenase, *F* forward, *R* reverse

### Cell culture and transfection

Human bladder cell lines T24 and RT4, normal bladder epithelial cell line SV-HUC-1, and HEK-293T cells were acquired from ATCC (Manassas, USA). Cells were cultivated in Dulbecco's modified Eagle's medium with 10% fetal bovine serum (FBS, all from Gibco Company, Grand Island, NY, USA) at 37 °C with 5% CO_2_. The miR-381 mimic and BMI1-overexpressing vectors (BMI1-OE) were synthesized by GenePharma Co., Ltd. (Shanghai, China). To minimize the off-target effects, 3 RNA fragments were loaded into the vectors and transfected into cells. Then cells were plated into 24-well plates once the cell confluence reaching 70%. miR-381 mimic and BMI1-OE vectors were transfected into T24 and RT4 cells in line with the protocols of the Lipofectamine 2000 (Invitrogen). After 6 h, the fluorescence expression in cells was observed under a fluorescence microscope (Olympus Optical Co., Ltd, Tokyo, Japan). In addition, 50 μmol/L Rho/Rock-specific antagonist, Y-27632 (MedChemExpress, Monmouth Junction, NJ, USA) [11], was further administrated into cells overexpressing BMI1. Cells transfected with an equal volume of DMSO were set as control. The cells were used for following experiments 48 h later.

### Cell proliferation by the cell counting kit-8 (CCK-8) method

A total of 100 μL cell suspension was loaded on 96-well plates and pre-incubated for 24 h. Next, each well was loaded with 10 μL CCK-8 reagent, and then the plates with cells were further incubated for 12, 24, 48 and 72 h. Then the optical density at 450 nm was evaluated using a microplate reader (SoectraMax iD5, Molecular Devices, USA). The CCK-8 kit was from Dojindo Laboratories (Kumamoto, Japan).

### Cell migration by scratch test

Guide lines were produced on the back side of 6-well plates at a 1-cm interval, and 5 × 10^5^ cells were added into the plates. On the second day, a pipette was used to produce a scratch perpendicular to the back side guide lines, and the scratched cells were washed away by phosphate buffer saline (PBS). Then the plates were loaded with serum-free medium and cultured at 37 °C with 5% CO_2_. Twenty-four hours later, the cells were observed and photographed under a microscope (CX22, Olympus). The scratch area was analyzed using the Image J software (version 1.48, NIH, USA). Relative migration distance (%) = (0 h scratch area—24 h scratch area)/0 h scratch area [12].

### Cell invasion by transwell assay

Matrigel was diluted in serum-free DMEM, and then 100 μL Matrigel was loaded onto each apical chamber at 37 °C for 4 h. Then, cells were resuspended in 10% FBS to 5 × 10^5^ cells/mL, and each apical chamber was loaded with 200 μL cell suspension, while each basolateral chamber was loaded with 600 μL cell medium (containing 5 μg/mL fibronectin). Then the transwells were incubated at 37 °C for 20 h, and the non-invaded cells in the apical chambers were removed, while the invaded cells in each well were stained with 500 μL 0.1% crystal violet at 37 °C for 30 min. Then the number of invaded cells were observed and counted under the microscope with 5 random fields included.

### Cell apoptosis by flow cytometry

In short, 27 mL ddH_2_O was used to dilute 3 mL binding buffer (10 ×). Cells in each group were resuspended in 1 mL 1 × binding buffer, centrifuged at 300 g for 10 min to discard the supernatant, and resuspended in 1 mL 1 × binding buffer again till a density of 1 × 10^6^ cells/mL. Next, each tube was loaded with 100 μL cells and then 5 μL Annexin V-fluorescein isothiocyanate (FITC) for 10 min of reaction, and then with 5 μL propidium iodide (PI) in the dark for 5 min. The apoptosis was determined using an Annexin V-FITC/PI Apoptosis Kit (Solarbio Science & Technology Co., Ltd., Beijing, China) and run on a flow cytometer (Attune Nxt, Thermo Fisher) within an hour.

### Xenograft tumors in nude mice

A total of 48 specific-pathogen-free nude mice (4–6 weeks old, 20 ± 2 g) purchased from Vital River Laboratory Animal Technology Co., Ltd. (Beijing, China) were randomly allocated into 6 groups, 8 in each. Then, each mouse was subcutaneously injected with 4 × 10^6^ T24 or RT4 cells with stable transfection of miR-381 mimic or mimic control, or cells with transfection of BMI1-OE and vector control. The volume (V) of xenograft tumors was calculated as the following formula every 7 days: V = (L × W^2^)/2, where ‘L’ indicates the length while ‘W’ indicates the width [13]. On the 28^th^ day after cell implantation, mice were euthanized by overdose of pentobarbital sodium (150 mg/kg), and the tumors were taken out and weighed.

### Dual-luciferase reporter gene assay

The binding relationship between miRNA and mRNA was predicted on StarBase (http://starbase.sysu.edu.cn/). The 3′UTR sequence of BMI2 and miR-381 was inserted into pMIR-REPORT™ luciferase reporter vector (Thermo Fisher) using Lipofectamine 3000 (Invitrogen) and then transfected into HEK293T cells. Cells were lysed 24 h later, and the luciferase activity was determined using a Dual-Luciferase Reporter Assay System (Promega Corporation, WI, USA) [14]. Three independent experiments were performed.

### Western blot analysis

Radio-Immunoprecipitation assay cell lysis buffer (Amresco Inc., Texus, USA) was used to collect total protein. In brief, single cell suspension was centrifuged at 800 g at 4 °C for 5 min with the supernatant discarded. Then the cells were ice-bated in a fivefold volume of lysis buffer for 10 min, and then centrifuged at 12,000 g at 4 °C for 10 min to collect the supernatant. The collected protein was run on SDS-PAGE and transferred to polyvinylidene fluoride membranes (EMD Millipore). Subsequently, the membranes were blocked with 5% skimmed milk and then incubated with the primary antibodies against RhoA (1:5000, ab187027), p-RhoA (1:1000, ab41435), ROCK2 (1:5000, ab71598) and β-actin (1:1000, ab8227) (Abcam Inc., Cambridge, MA, USA) at 4 °C for 16 h, and then with the secondary antibody (1:3000, ab205718) at room temperature for 2 h. The immunoblotting image were visualized using the Image J software (Version 1.8.0, National Institute of Health, USA). Three independent experiments were performed.

### Statistical analysis

SPSS 22.0 (IBM Corp. Armonk, NY, USA) was used for data analysis. Data were in normal distribution according to Kolmogorov–SmiRnov test and are exhibited as mean ± standard deviation (mean ± SD). Differences in multiple groups were analyzed using one-way or two-way analysis of variance (ANOVA). Tukey’s multiple comparisons test was used for the post-hoc test after ANOVA analysis. Survival curve was drawn using the Kalpan-Meier method and analyzed using the log rank test. *p* was obtained by two-tailed tests and *p* < 0.05 was regarded statistically significant.

## Results

### Poor miR-381 expression in BCa patients indicates unfavorable prognosis

**Fig. 1:**
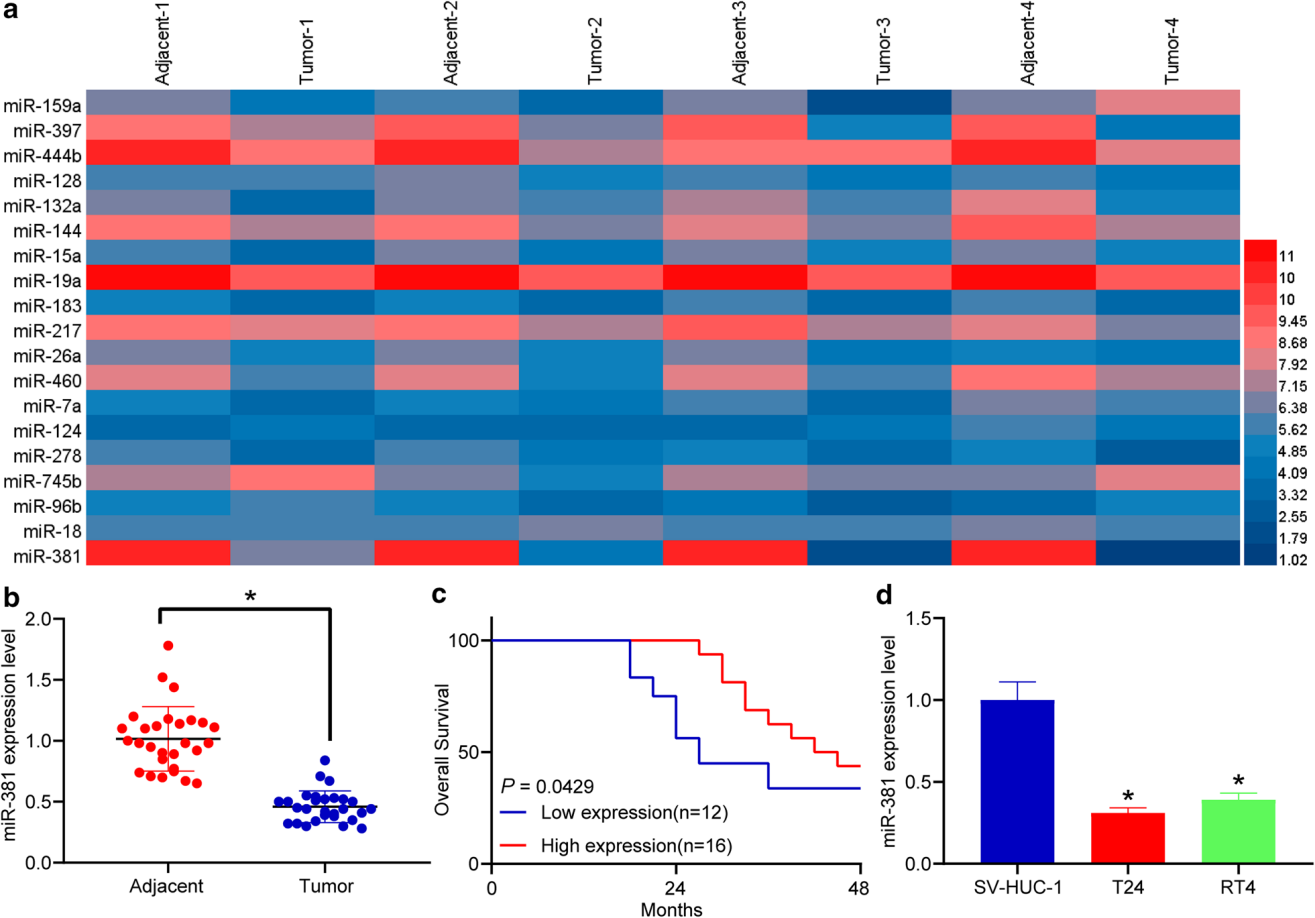
Poor miR-381 expression in BCa patients indicates unfavorable prognosis. **a** differentially expressed miRNAs between BCa tissues and the paired adjacent tissues determined by microarray analysis (*p* < 0.05, |logFC|≥ 1.5); **b** miR-381 expression in the BCa and paired adjacent tissues determined by RT-qPCR (*p < 0.05, two-way ANOVA); **c** survival analysis of BCa patients with different level of miR-381; **d** miR-381 expression in BCa cells (T24 and RT4) and in normal bladder epithelial cell line SV-HUC-1 determined by RT-qPCR (*p < 0.05, one-way ANOVA); Repetition = 3

The tumor tissues and paired adjacent tissues from 4 BCa patients (T1 to T4) were used for miRNA microarray analysis. It was found that miR-381 was the greatest down-regulated one in tumor tissues as compared to the paired normal ones (Fig. 1a). Then, the RT-qPCR results validated that miR-381 was significantly poorly expressed in the tumor tissues in all the included BCa patients (Fig. 1b). According to the average miR-381 expression (0.46), the patients were allocated into high-expression group (n = 16) and low-expression group (n = 12). The follow-up study data suggested that patients with higher miR-381 had relatively higher survival rates and longer lifetime (Fig. 1c). In addition, we also determined miR-381 expression in cell levels, and it is noteworthy that miR-381 expression was much lower in BCa cell lines T24 and RT4 than that in normal bladder epithelial cells (Fig. 1d).

### miR-381 inhibits malignant behaviors of BCa cells

**Fig. 2:**
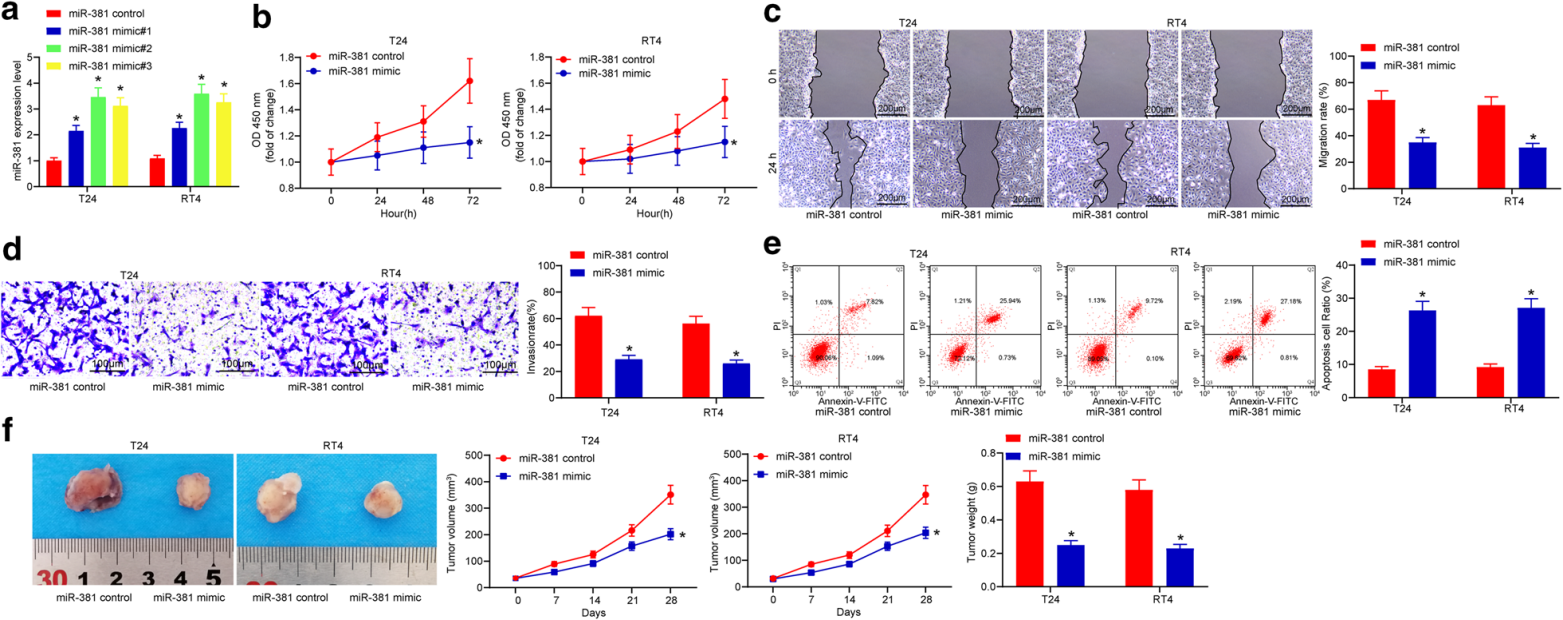
miR-381 inhibits malignant behaviors of BCa cells. **a** miR-381 expression in cells after miR-381 mimic transfection detected by RT-qPCR, and it was found that miR-381 mimic#2 showed the best efficacy (*p < 0.05, two-way ANOVA); **b** optical density at 450 nm measured by two-way ANOVA (*p < 0.05, two-way ANOVA); **c** migration rate of each group of cells at the 24th h (*p < 0.05, two-way ANOVA); **d** invasion rate of each group of cells measured by transwell assay (*p < 0.05, two-way ANOVA); **e** apoptosis rate of cells determined by flow cytometry (*p < 0.05, two-way ANOVA); **f** tumor volume change and weight on the 28th day after implantation of cells transfected miR-381 mimic (*p < 0.05, two-way ANOVA); Repetition = 3

To further figure out the roles of miR-381 in BCa development, up-regulation of miR-381 was introduced in both T24 and RT4 cell lines by administrating miR-381 mimic or mimic control (Fig. 2a). Then, we found that miR-381 mimic treatment led to a significant decline in proliferation in both cells (Fig. 2b). Accordingly, cells with increased miR-381 expression presented decreased migration distance (Fig. 2c) and reduced number of invaded cells (Fig. 2d). In addition, it was found that miR-381 mimic led to increased cell apoptosis (Fig. 2e). As for in vivo experiments, the implantation of cells with miR-381 mimic led to a significant decline in tumor growth in mice, and the tumor weight was decreased on the 28^th^ day (n = 8) (Fig. 2f).

### miR-381 targets BMI1 to inactivate the Rho/Rock pathway

**Fig. 3:**
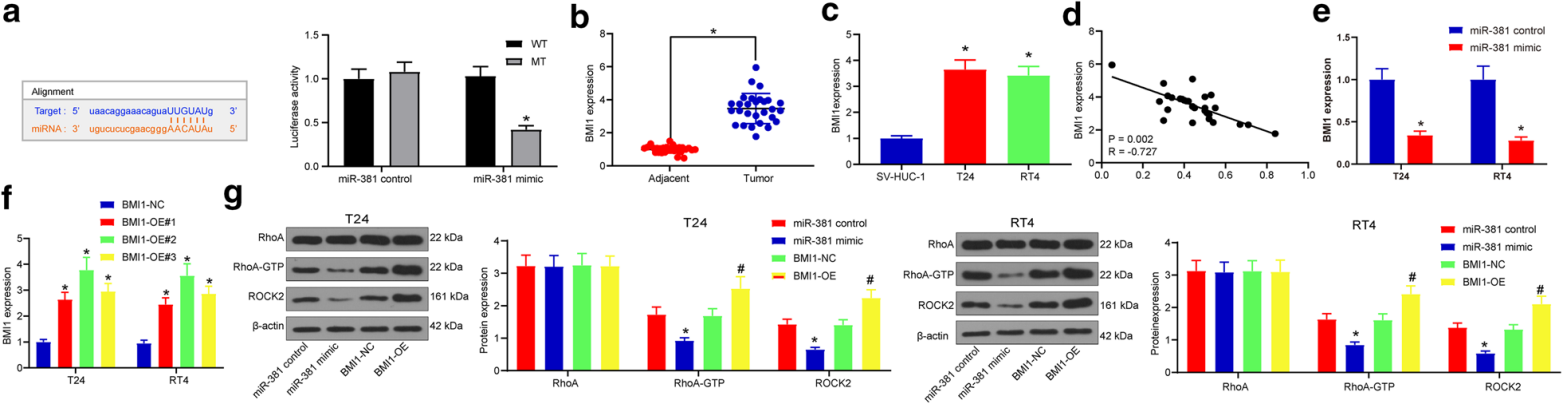
miR-381 targets BMI1 to inactivate the Rho/Rock pathway. **a** target relationship between miR-381 and BMI1 predicted on StarBase (http://starbase.sysu.edu.cn/) and validated through a dual luciferase reporter assay (*p < 0.05, two-way ANOVA); **b** BMI1 expression in BCa tissues determined by RT-qPCR (*p < 0.05, two-way test); **c** BMI expression in BCa cell lines (T24 and RT4) and in normal bladder epithelial cells determined by RT-qPCR (*p < 0.05, one-way ANOVA); **d** correlation analysis between miR-381 and BMI1 expression in BCa tumor tissues; **e** BMI1 expression in T24 and RT4 cells after miR-381 mimic transfection measured by RT-qPCR (*p < 0.05, two-way ANOVA); **f** BMI1 expression in BCa tumor tissues after BMI1-OE vector transfection detected by RT-qPCR (*p < 0.05, two-way ANOVA). **g** protein levels of ROCK2, RhoA and RhoA-GFP (phosphorylated RhoA) in each group of cells measured by western blot analysis (*p < 0.05, two-way ANOVA); Repetition = 3

We next explored the potential downstream molecules involved. We first predicted BMI1 as a target of miR-381 on a computer-based bioinformation system, and then had the binding relationship between miR-381 and BMI1 validated through a luciferase assay (Fig. 3a). Then, it was found that BMI1 expression was increased in tumor tissues as compared to the adjacent tissues (Fig. 3b), and similarly, the BMI1 expression was also higher in BCa cell lines than that in bladder epithelial cells (Fig. 3c). The BMI1 expression in the tumor tissues of BCa patients was negatively correlated with miR-381 expression (Fig. 3d). Importantly, BMI1 expression in BCa cells was decreased upon miR-381 upregulation (Fig. 3e). BMI1-OE vectors were administrated into T24 cells and RT4 cells, and BMI1-OE#2 presented a best overexpressing efficiency (Fig. 3f). Intriguingly, the Rho/ROCK signaling pathway has been noted to be significantly associated with the aggressiveness of BCa [15]. After miR-381 control, miR-381 mimic, BMI1-NC or BMI1-OE vector transfection, the protein levels of RhoA and ROCK2 were detected. It was found that miR-381 mimic inhibited the ROCK2 expression and RhoA phosphorylation while BMI1-OE led to increased activation of the Rho/ROCK signaling pathway (Fig. 3g). The above results indicated that miR-381 could target BMI1 to inactivate the Rho/Rock pathway.

### Overexpression of BMI1 promotes BCa progression

**Fig. 4:**
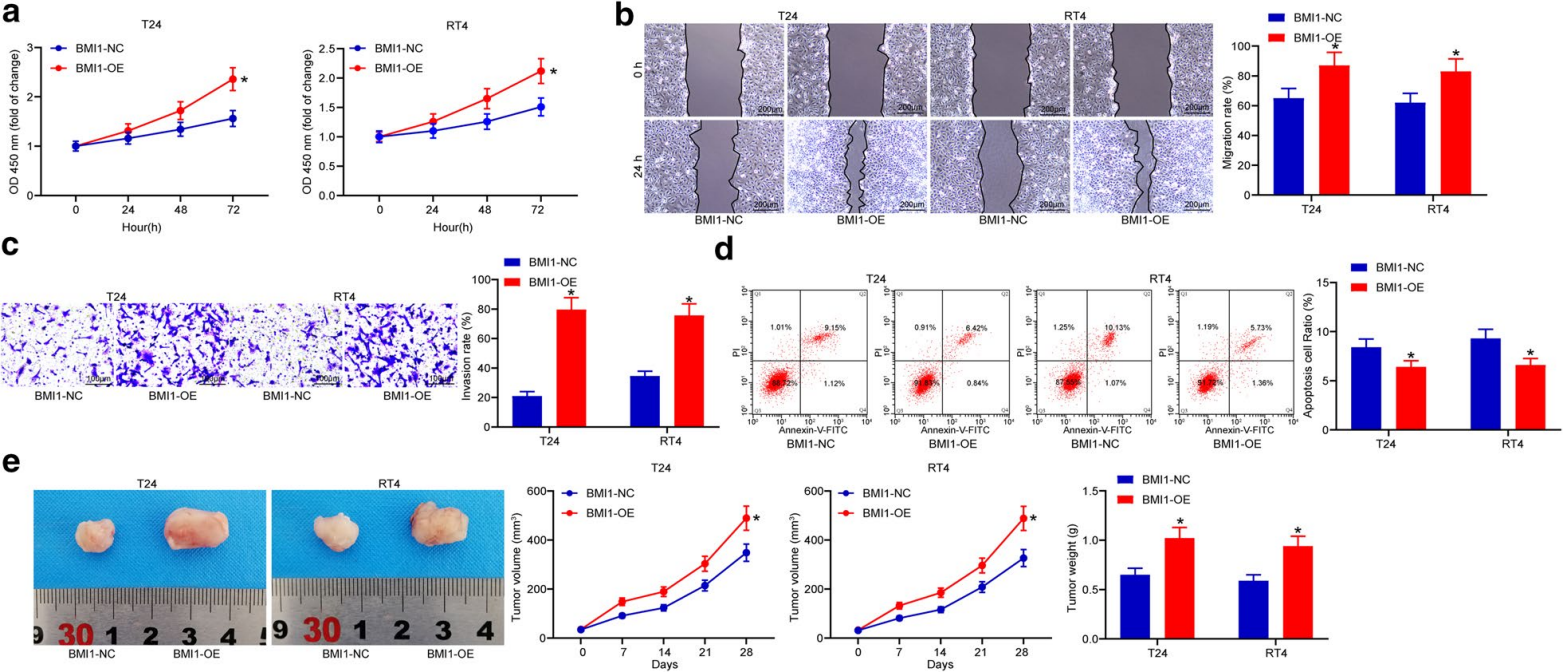
Overexpression of BMI1 promotes BCa progression. **a** optical density at 450 nm of each group of cells according the CCK-8 method (*p < 0.05, two-way ANOVA); **b** migration distance at the 24^th^ hour measured by the scratch test (*p < 0.05, two-way ANOVA); **c** number of invaded cells at the 24^th^ hour determined by the transwell assay (*p < 0.05, two-way ANOVA); **d** number of PI-Annexin V-double positive cells detected by flow cytometry (*p < 0.05, two-way ANOVA); **e** tumor growth rate change and weight on the 28th day in nude mice (n = 8) (*p < 0.05, two-way ANOVA); Repetition = 3

To further identify the exact roles of BMI1 in BCa pathogenesis, overexpression of BMI1 was artificially up-regulated in T24 and RT4 cells. BMI1-OE vector led to an increase in cell proliferation (Fig. 4a). Likewise, the migration rate (Fig. 4b) and invasion rate (Fig. 4c) of cells was notably increased following BMI1 overexpression. In addition, overexpression of BMI1 reduced the apoptosis rate in both cell lines (Fig. 4d). As for in vivo, we found cells transfected with BMI1-OE led to significantly increased tumor growth rate and weight in nude mice (n = 8) (Fig. 4e). These results identified BMI1 presented a reverse regulating role in BCa cells as relative to miR-381.

### BMI1 partially blocks the role of miR-381

**Fig. 5:**
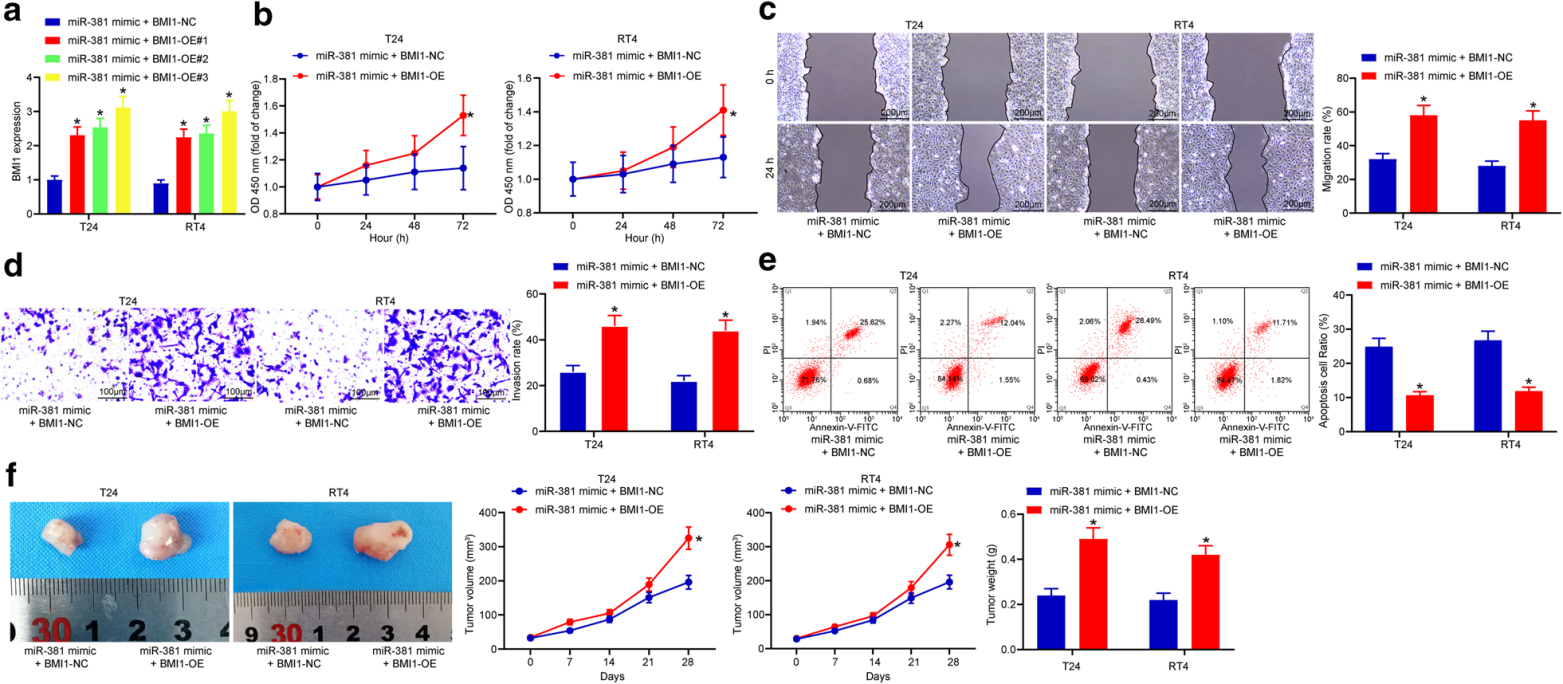
BMI1 partially blocks the role of miR-381. **a** cells pre-transfected with miR-381 mimic were further transfected with BMI1-OE vectors, and it was found that BMI1-OE#3 presented the best overexpressing efficiency (*p < 0.05, two-way ANOVA); **b** optical density at 450 nm of each group of cells according the CCK-8 method (*p < 0.05, two-way ANOVA); **c** migration distance at the 24th hour measured by the scratch test (*p < 0.05, two-way ANOVA); **d** number of invaded cells at the 24th h determined by the transwell assay (*p < 0.05, two-way ANOVA); **e** number of PI-Annexin V-double positive cells detected by flow cytometry (*p < 0.05, two-way ANOVA); **f** tumor growth rate change and weight on the 28th day in nude mice (n = 8) (*p < 0.05, two-way ANOVA); Repetition = 3

To further confirm the involvement of BMI1 in the miR-381-mediated events. Overexpression of BMI1 was introduced in cells pre-transfected with miR-381 mimic (Fig. 5a). It was found that further overexpression of BMI1 partially recovered cell proliferation (Fig. 5b), cell migration (Fig. 5c) and cell invasion (Fig. 5d) that were suppressed by miR-381 mimic. In addition, the miR-381-triggerred cell apoptosis was inhibited when BMI1 was further overexpressed (Fig. 5e). Accordingly, cells transfected miR-381 mimic and BMI-OE vector led to an increased tumor growth rate in nude mice compared to cells with miR-381 mimic alone (Fig. 5f). These findings suggested that BMI1 partially antagonized the effects of miR-381 mimic.

### Inhibition of Rho/ROCK signaling blocks the functions of BMI1

**Fig. 6:**
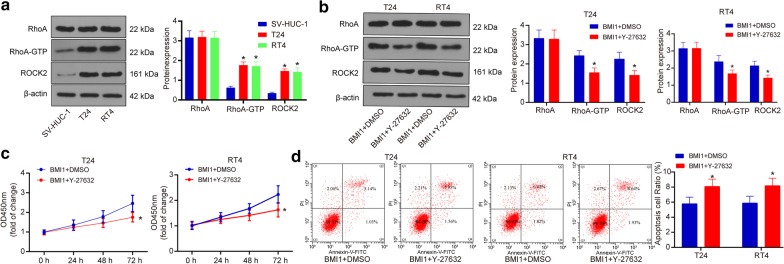
Inhibition of Rho/ROCK signaling blocks the functions of BMI1. **a**, **b** Protein levels of ROCK2, RhoA and RhoA-GFP (phosphorylated RhoA) in each group of cells measured by western blot analysis (*p < 0.05, two-way ANOVA); **c** optical density at 450 nm of each group of cells according the CCK-8 method (*p < 0.05, two-way ANOVA); **d** number of PI-Annexin V-double positive cells detected by flow cytometry (*p < 0.05, two-way ANOVA). Repetition = 3

To further validate the involvement of the Rho/ROCK signaling in the above events, we first determined the Rho/ROCK2 activity in T24, RT4 and SV-HUC-1 cell lines. It was found that the phosphorylation of RhoA and protein level of ROCK2 was increased in BCa cell lines (Fig. 6a). Then, a Rho/ROCK-specific antagonist, Y-27632, was administrated into cells overexpressing BMI1, after which the phosphorylation of RhoA and protein level of ROCK2 was decreased (Fig. 6b). In addition, the CCK-8 and flow cytometry results showed that the increased cell growth and decreased cell apoptosis by BMI1 were blocked by Y-27632 (Fig. 6c, d), indicating this pathway is at least partially responsible for the BMI1-medeated cell growth.

## Discussion

Treatment for BCa, especially for the invasive type MIBC remains great challenge owing to high recurrence following surgical resection and drug administration. Aside from the unfavorable prognosis, the huge economic cost in lifetime surveillance with periodic cystoscopy and evaluation of recurrence rate brings considerable burden to BCa patients [16–18]. It has been an emerging consensus that aberrant expression of some miRNAs, either extremely high or poor expression, is closely correlated with BCa pathogenesis [19]. Here, the current study evidenced that miR-381 could serve as a cancer suppressor in BCa with the involvement of BMI1 downregulation and the following Rho/ROCK inactivation.

The initial finding was that miR-381 was poorly expressed in the tumor tissues in BCa patients and was positively correlated with patient prognosis, preliminarily indicating a beneficiary role of miR-381 in BCa prognosis. miRNAs have been emerged as non-invasive biomarkers for BCa detection [20, 21] or even potential treating targets in clinical practice [22]. For instance, miR-3622a was noted to promote proliferation and invasion capacities of BCa through binding to LASS2 [12]. On the other hand, some miRNAs have been documented to exert suppressing functions in BCa. miR-502-5p, for example, has been recently found to suppress the malignant behaviors of BCa cells through the different miRNA targets including CCND1, NOP14 and DNMT3B [23]. Similar trends have also been seen in miR-124-3p [24] and miR-203a [25]. As for miR-381, it has been particularly suggested to play critical functions in overcoming cisplatin resistance in breast cancer treatment [26–28]. Likewise, its anti-tumor role has been increasingly revealed in other malignancies such as in cervical cancer [29] and in prostate cancer [30] through the different mRNA targets. Here our study found that miR-381 was poorly expressed in BCa cells. Overexpression of miR-381 inhibited the malignant behaviors of BCa T24 and RT4 cell lines, presenting as decreased cell viability, reduced cell migration and invasion, and increased cell apoptosis. In addition, similar results were found in animal experiments, where cells with stable miR-381 overexpression contributed to a significant inhibition in tumor growth in volume and weight regards.

Second, the findings above triggered us to further explore the downstream molecules of miR-381. A computer-based online prediction system suggested BMI1 as a target mRNA of miR-381, and this binding relationship was further validated by a luciferase assay. As a member of the polycomb group family, BMI1, owning stem cell characteristics, is prone to participate in the onset and development of tumors and is linked to tumor metastasis, recurrence and chemo-resistance as well [31]. In addition, BMI1 is well-known to interact with another EMT and stemness promoter, TWIST1 [32, 33]. Not surprisingly, silencing of BMI1 has been suggested as a promising therapeutic target in many human cancer therapies [34, 35]. Here, our study found that artificial overexpression of BMI1 led to increased cell proliferation, migration, invasion and resistance to death in both T24 and RT4 cells. The involvement of BMI1 in BCa pathogenesis has been relatively largely studied. Its knockdown was noted to inhibit bladder cell growth, self-renewal and progression, and several upstream mediators of BMI1 such as miR-139-5p [36] and miR-200c [37] were identified. In the current paper, in order to further validate that BMI1 silencing holds accountable for the miR-381-mediated events, rescue experiments were performed where cells pre-transfected with miR-381 were further transfected with BMI1-OE. It was found that the malignant behaviors of cells inhibited by miR-381 mimic were partially recovered following BMI1 overexpression.

The Rho/ROCK signaling is well-known for its implication in cytoskeletal reorganization, which is elemental for cell migration and metastasis [38]. Specifically, this signaling was documented to be related to the invasion and metastasis and malignancy in BCa [15]. Here, our study identified that the phosphorylation of RhoA and ROCK2 expression was notably inhibited by miR-381, while overexpression of BMI1 led to reversed trends. A Rho‑associated kinase inhibitor, Y-27632, has been suggested to inhibit the migration and metastasis of BCa [11]. Here, we further noticed that Y-27632 blocked the promoting roles of BMI1 in BCa cell growth. Therefore, it can be inferred that the Rho/ROCK pathway inactivation is possibly implicated in the miR-381/BMI1-mediated events.

## Conclusion

**Fig. 7:**
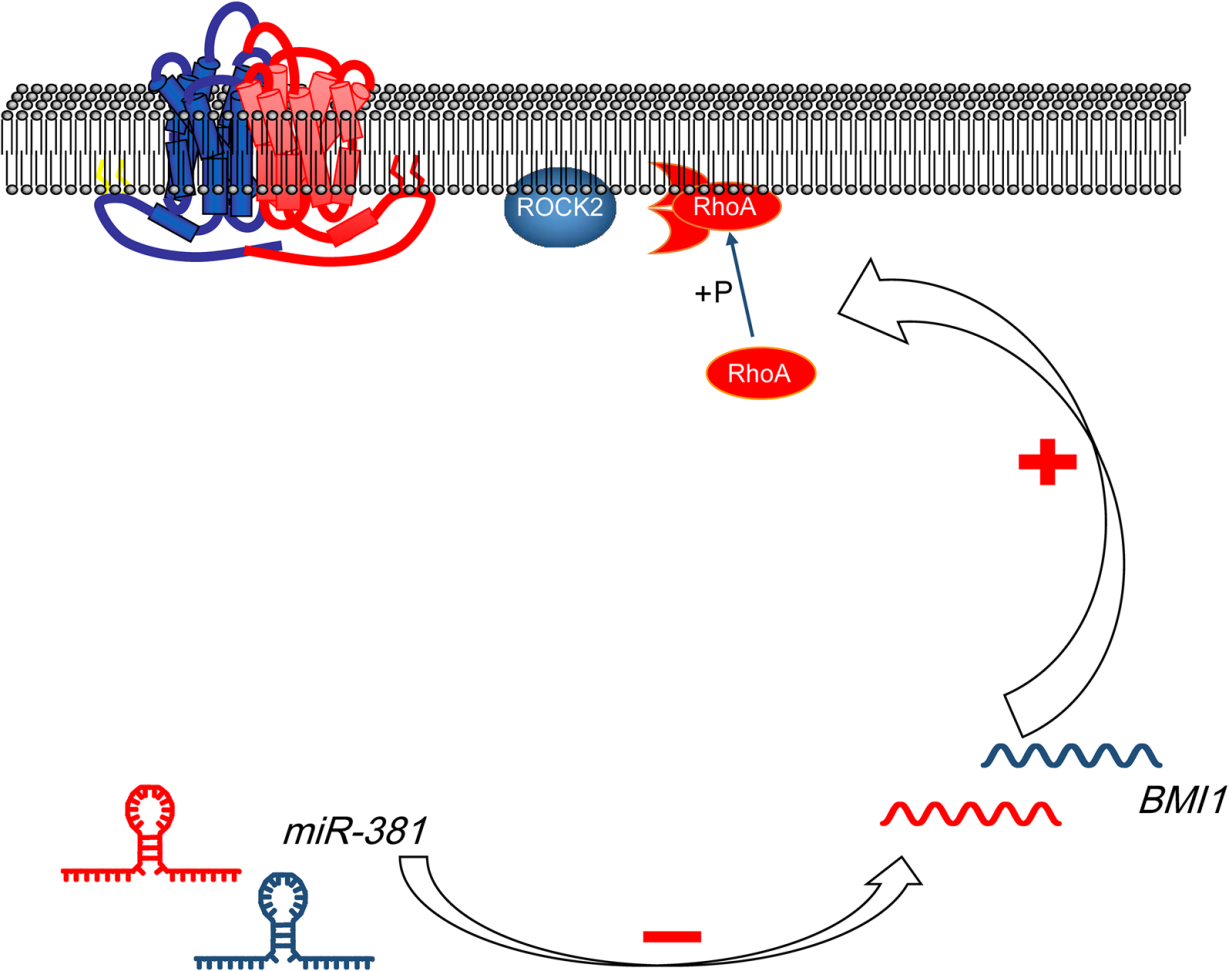
A diagram for the molecular mechanism. In BCa cells, miRNA-381 directly binds to BMI1 expression to inhibit RhoA phosphorylation and ROCK2 expression, leading to suppressed malignant behaviors of bladder cells

To sum up, the present study evidenced that miR-381 could inhibit BCa progression through inhibiting BMI1, presenting as decreased cell proliferation, migration, invasion and resistance to death, as well as tumor growth in vivo. The Rho/ROCK pathway was found to be at least partially responsible for the above events (Fig. 7). However, this is a pre-clinical study with limited sample size, and the regulatory network by which BMI1 regulate the Rho/ROCK requires further investigation. Still, the findings of the present study need further validation. We hope these findings may offer novel insights into BCa treatment.

## Data Availability

All the data generated or analyzed during this study are included in this published article. And the identifying/confidential patient data are not be shared.

## Abbreviations

ANOVA: Analysis of variance
BMI1: B-Cell-specific Moloney murine leukemia virus insertion region 1
CCK-8: Cell counting kit-8
EMT: Epithelial-mesenchymal transition
FBS: Fetal bovine serum
FITC: Fluorescein isothiocyanate
mean ± SD: Mean ± standard deviation
MIBD: Muscle invasive bladder cancer
miRNA: MicroRNA
NMIBD: Non-muscle invasive bladder cancer
PBS: Phosphate buffer saline
PI: Propidium iodide
RT-qPCR: Reverse transcription quantitative polymerase chain reaction
TNM: Tumor-Node-Metastasis
3′UTR: 3′Untranslated region
